# Male and Female Discrepancies in Anxiety, Depressive Symptoms, and Coping Strategies among Orthognathic Patients: A Cross-Sectional Analysis

**DOI:** 10.3390/jcm12227161

**Published:** 2023-11-18

**Authors:** Robert Avramut, Serban Talpos, Camelia Szuhanek, Marius Pricop, Roxana Talpos, Tareq Hajaj, Nicoleta Nikolajevic-Stoican, Raluca Maracineanu, Roxana Ghircau-Radu, Malina Popa

**Affiliations:** 1 Doctoral School, “Victor Babes” University of Medicine and Pharmacy, Eftimie Murgu Square 2, 300041 Timisoara, Romania; robert.avramut@umft.ro (R.A.); nicoleta_stoican@yahoo.com (N.N.-S.); ralucazibileanu@yahoo.com (R.M.); 2 Discipline of Oral and Maxillo-Facial Surgery, Faculty of Dental Medicine, “Victor Babes” University of Medicine and Pharmacy Timisoara, Revolutiei Boulevard 9, 300041 Timisoara, Romania; pricop.marius@umft.ro; 3 Discipline of Orthodontics, Faculty of Dental Medicine, “Victor Babes” University of Medicine and Pharmacy Timisoara, Revolutiei Boulevard 9, 300041 Timisoara, Romania; cameliaszuhanek@umft.ro; 4 Discipline of Odontotherapy-Endodontics, Faculty of Dental Medicine, “Victor Babes” University of Medicine and Pharmacy, Eftimie Murgu Square 2, 300041 Timisoara, Romania; roxanaclinci@yahoo.com; 5 Discipline of Prostheses Technology and Dental Materials, Faculty of Dental Medicine, “Victor Babes” University of Medicine and Pharmacy, Eftimie Murgu Square 2, 300041 Timisoara, Romania; tareq.hajaj@umft.ro; 6 Department of Pediatric Dentistry, Faculty of Dental Medicine, “Victor Babes” University of Medicine and Pharmacy, Eftimie Murgu Square 2, 300041 Timisoara, Romania; popa.malina@umft.ro; 7 Faculty of Dental Medicine, “Vasile Goldis” Western University of Arad, Revolutiei Boulevard 94-96, 310025 Arad, Romania; radu.roxana@uvvg.ro

**Keywords:** gender differences, anxiety, depression, coping behavior

## Abstract

With an increasing understanding of the differences between men and women’s psychological experiences, this study aimed to probe the sex-based differences in anxiety, depressive symptoms, and coping strategies among orthognathic patients. The study hypothesis was that female patients would show higher levels of anxiety and depressive symptoms than males, and that coping mechanisms would differ between male and female sexes. A cross-sectional design was adopted, examining orthognathic patients from the Department of Oral and Maxillo-Facial Surgery at the Emergency Clinical Municipal Hospital in Timisoara, Romania, from 2020 to 2023. Eligible participants (18+ years with no prior orthognathic treatment) completed a comprehensive online questionnaire 6 weeks before scheduled surgery. This was composed of validated self-report instruments comprising the SF-36, GAD-7, and the PHQ-9, and the COPE-60, along with additional sociodemographic data. Of the 127 orthognathic patients analyzed (68 men and 59 women, aged 18 to 65 years, mean age 32), men rated their physical health status slightly better on the SF-36 scale. However, the most notable difference was in mental health, with females scoring higher on both the PHQ-9 (indicative of depression) and the GAD-7 (indicative of anxiety) scales. Specifically, female participants exhibited average PHQ-9 scores 1.8 points higher and GAD-7 scores 1.5 points higher than their male counterparts. Coping mechanisms also varied: 42% of male patients primarily employed “Disengagement” strategies, while 58% of females predominantly used “Engagement” and “Emotion Focused” strategies. Emotion-focused coping was associated with a 1.6-fold increased risk of depressive symptoms. Sex differences play a crucial role in the psychological experiences of orthognathic patients, evident in anxiety and depression levels, perceived health status, and coping strategies. This underlines the importance of sex-tailored psychological support in the preoperative phase for orthognathic surgery patients.

## 1. Introduction

Orthognathic surgery, often referred to as corrective jaw surgery, is undertaken to rectify conditions and deformities related to the jaw and face [1,2]. These conditions often have both functional and aesthetic implications, leading to difficulties in biting, chewing, speaking, and even breathing [3,4]. Beyond the functional issues, facial skeletal deformities can be associated with psychosocial distress owing to societal norms and perceptions regarding facial appearance [5,6]. Therefore, orthognathic patients often face a dual burden of managing the physical implications of their conditions, as well as the psychological challenges associated with perceived aesthetic shortcomings [7].

The patient’s sex has been identified as an influencing factor in the prevalence and manifestation of anxiety and depressive disorders in the general population [8,9]. Women, for instance, are almost twice as likely as men to suffer from anxiety disorders, and similar discrepancies exist for depressive disorders [10]. Furthermore, the coping strategies employed by men and women in dealing with stress and adversity have been found to differ [11]. While women tend to utilize emotion-focused coping strategies like seeking emotional support, men lean towards problem-focused coping mechanisms such as active problem solving [12].

In the context of orthognathic surgery, where patients are navigating both functional challenges and the psychological complexities associated with facial appearance, it becomes crucial to understand male–female discrepancies in experience and coping mechanisms [13,14]. Preliminary research has suggested that orthognathic patients might experience heightened levels of anxiety and depressive symptoms, with these manifestations possibly differing between sexes [15,16]. However, a comprehensive examination of these disparities and the associated coping strategies in the orthognathic patient population remains, more in terms of male–female disparities, limited [17,18,19]. In this context, one meta-analysis investigated the psychological effects of orthognathic surgery on patients with dentofacial abnormalities. It included 37 studies and found significant improvements in various psychological domains, such as depression and self-esteem, after treatment. The findings underscore the role of surgeons and orthodontists in managing patient expectations and considering psychological well-being in treatment plans [20].

The importance of understanding these sex discrepancies is multi-fold. From a clinical perspective, tailoring pre-operative and post-operative interventions based on the unique psychological needs of male and female patients might enhance their surgical outcomes and overall well-being. From a societal standpoint, this knowledge can assist in dispelling stereotypes and myths surrounding facial deformities, thus fostering a more inclusive and empathetic environment for individuals undergoing orthognathic procedures.

Recognizing the potential implications of patient’s sex on the psychological experiences of orthognathic patients, this study aims to provide a detailed cross-sectional analysis of variations by sex regarding anxiety, depressive symptoms, and coping strategies among this patient population. We hypothesize that (1) female orthognathic patients will report higher levels of anxiety and depressive symptoms compared to their male counterparts, and (2) there will be discernible differences in the coping strategies employed by male and female patients. The primary objectives of this study are to elucidate the sex-based disparities in psychological symptoms and coping mechanisms among orthognathic patients and offer insights for personalized patient care.

## 2. Materials and Methods

### 2.1. Research Design and Ethical Considerations

The study employed a cross-sectional research design to explore the male–female discrepancies in anxiety, depressive symptoms, and coping strategies among orthognathic patients. Patients were recruited from the Emergency Clinical Municipal Hospital in Timisoara, Romania, during a three-year span (2020–2023), at the Department of Oral and Maxillo-Facial Surgery. In adherence to the strictest ethical standards, the study was approved by the hospital’s Ethics Committee for Research, ensuring compliance with international research guidelines and the principles specified in the Declaration of Helsinki.

The scope of this study was confined to examining the biological-sex differences (male and female) in anxiety, depressive symptoms, and coping strategies among orthognathic patients, as indicated by the participants’ self-reported sex at birth. The current study did not explore gender identity as a broader social construct, which includes cultural, behavioral, and personal identification factors.

### 2.2. Inclusion Criteria and Definitions

Patients completed the online questionnaire within 6 weeks prior to their scheduled surgery date and each questionnaire was coded to protect confidentiality. The questionnaire incorporated four validated and psychometrically tested self-report instruments that evaluated anxiety, depressive symptoms, and coping strategies. Additional sections gathered sociodemographic information, type of planned surgery, and satisfaction with the information provided by the clinical team. The participant selection began by liaising with treating surgeons to select potential candidates ready for orthognathic surgery. Eligible patients were adults aged 18 and above who had not undergone previous orthognathic treatment or ‘surgery first’ procedures. Exclusion criteria encompassed patients without explicit consent, those with craniofacial syndromes, individuals who had previously undergone orthognathic treatment, ‘surgery first’ patients, and those with incomplete questionnaire responses.

After meeting the inclusion criteria and agreeing to participate, patients were provided with a link to the online questionnaire. The participants were given clear instructions on how to complete the questionnaire and were assured of the confidentiality of their responses. They were also informed of their right to withdraw from the study at any point without any implications for their treatment.

### 2.3. Variables

The cross-sectional assessment aimed to evaluate potential sex-based differences in anxiety levels, depressive symptoms, and coping mechanisms among the orthognathic patient population. Variables assessed included the patient’s age, sex, socio-economic background, and type of orthognathic procedure they were scheduled to undergo. A major emphasis was placed on their psychological experiences and coping strategies in anticipation of the surgery. By examining these variables, the study aimed to provide insights into sex disparities among orthognathic patients. In line with data protection guidelines, all data collated were anonymized.

### 2.4. Surveys Employed

To properly evaluate the participants’ experiences, four widely recognized tools were administered. The SF-36 Health Survey [21] was used to measure the quality of life, encompassing eight scales: physical functioning, role limitations due to physical health, pain, general health, energy/fatigue, social functioning, role limitations due to emotional health, and emotional well-being. For the assessment of anxiety and depression, the GAD-7 (Generalized Anxiety Disorder 7-item scale) [22] and PHQ-9 (Patient Health Questionnaire-9) were used, respectively [23]. The GAD-7 quantifies anxiety symptoms, and the PHQ-9 evaluates the severity of depressive symptoms. Lastly, the COPE-60 survey [24] was conducted to determine the coping strategies employed by both male and female participants. The COPE-60 is broken down into different subscales, each representing different coping strategies (Disengagement, Engagement, Emotion Focused, and Problem Focused).

(a) Disengagement: This is a form of avoidance coping, where individuals detach themselves from the stressor or the associated emotions. A higher score in this subscale might indicate that a person tends to avoid dealing with the stressor.
(b) Engagement: This is an approach coping strategy, where individuals actively confront and engage with the stressor. A higher score here might mean that the individual tends to address stressors head-on.
(c) Emotion Focused: This type of coping concerns managing emotional distress rather than the actual problem or situation causing the distress. Higher scores indicate that the individual frequently uses emotion-focused strategies like seeking emotional support or expressing feelings.
(d) Problem Focused: This strategy is about directly addressing the problem. Higher scores on this subscale mean that the individual prefers to take direct actions to resolve the stressor.

### 2.5. Statistical Analysis

Data management and analysis were conducted utilizing the statistical software SPSS version 26.0 (SPSS Inc., Chicago, IL, USA). The sample size was calculated based on a convenience sampling method, with a minimum of 80 respondents at a 95% confidence level and 10% margin of error. Normality tests were carried using the Kolmogorov–Smirnov test. Continuous variables were represented as mean ± standard deviation (SD), while categorical variables were expressed in terms of frequencies and percentages. To analyze the changes between more than two means of continuous variables, the Student’s *t*-test was utilized. The chi-square test was utilized to compare the proportions for the categorical variables. A multivariate regression analysis was performed to determine the risk factors for depression and depressive symptoms among orthognathic patients. A *p*-value threshold of less than 0.05 was set for statistical significance. All results were double-checked to ensure accuracy and reliability.

## 3. Results

By the end of the study period, the final cohort consisted of 127 orthognathic patients (68 men and 59 women) who met the inclusion criteria and completed the questionnaire in its entirety. The age distribution ranged from 18 to 65 years, with an average age of 32 years. The place of origin, classified as urban, was reported for 55.9% of male participants and 61.0% of female participants, with no significant difference observed (*p* = 0.558). The Charlson Comorbidity Index (CCI) greater than two was observed in 8.8% of men and 6.8% of women, and this difference was also not statistically significant (*p* = 0.669). The relationship status, categorized as single, was higher among women (49.2%) compared to men (36.8%), but the difference fell short of statistical significance (*p* = 0.159). In terms of employment, unemployment rates were slightly higher in men (16.2%) than in women (13.6%), although not statistically different (*p* = 0.683).

For malocclusion types, the distribution between men and women was also found to be statistically similar across types I, II, and III (*p* = 0.706). The choice between single-jaw and bimaxillary surgeries showed no significant sex-based preference, with 51.5% of men and 47.5% of women undergoing single-jaw surgeries and 48.5% of men and 52.5% of women undergoing bimaxillary surgeries (*p* = 0.651), as presented in Table 1.

**Figure 1:**
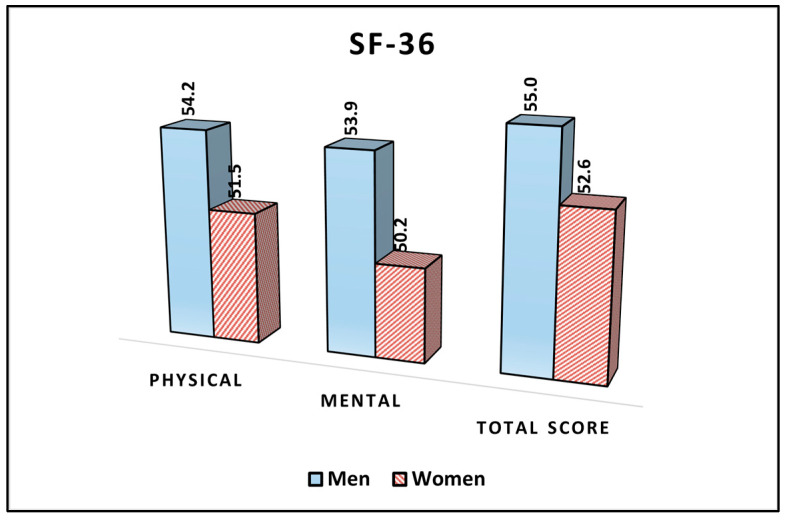
Analysis of the SF-36 questionnaire results stratified by patients’ sex.

In the physical domain of the survey, men reported an average score of 54.2, while women reported a slightly lower average score of 51.5. This difference between the patients’ sex was statistically significant (*p* = 0.049), suggesting that men, on average, rated their physical health status slightly better than women in the study population. For the mental domain, a more pronounced difference between genders was observed. Men reported an average score of 53.9, while women had an average score of 50.2. The difference in the mental health status between men and women was statistically significant (*p* = 0.003), indicating that men perceived their mental health status to be better than women in this sample. Regarding the total score of the SF-36 survey, there were no statistically significant differences (*p* = 0.083), as seen in Table 2 and Figure 1.

Regarding the GAD-7 survey, which evaluates symptoms of generalized anxiety disorder, men had an average score of 5.9, while women reported a higher average score of 7.1, showing a statistically significant difference (*p* = 0.037). Thus, it can be inferred that women in the study population, on average, exhibited more-pronounced symptoms of anxiety compared to their male counterparts.

**Figure 2:**
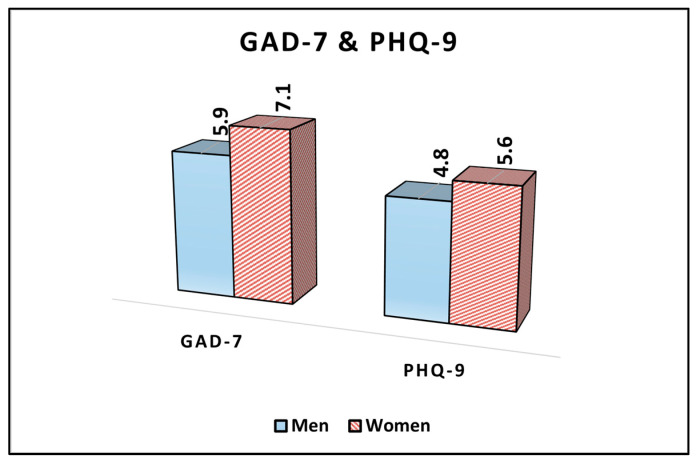
Analysis of the GAD-7 and PHQ-9 questionnaire results stratified by patients’ sex.

As for the PHQ-9, which assesses depressive symptoms, men recorded an average score of 4.8, whereas women had an average score of 5.6. Although women had a higher average score, indicating potentially more severe depressive symptoms, this difference between sexes was not statistically significant (*p* = 0.091), as described in Table 3 and Figure 2. Therefore, while there appeared to be a trend suggesting women might have slightly more severe depressive symptoms than men, this observation was not confirmed with statistical significance in the study cohort.

The COPE-60 assesses different domains of coping strategies, with higher scores signifying a greater likelihood of a patient employing a specific domain of coping strategy. Examining the results for “Disengagement” as a coping strategy, 61.8% of men, equivalent to 42 individuals, scored above the median. In contrast, a smaller percentage, 44.1% (equating to 26 individuals), of women scored above the median in this domain. This observed disparity between sexes was statistically significant (*p* = 0.046), suggesting that male orthognathic patients were more prone to employing disengagement as a coping mechanism compared to their female counterparts.

Regarding the “Engagement” strategy, only 26.5% of men scored above the median, while a considerably higher percentage of women, 50.8%, scored above the median, showing a significant difference (*p* = 0.004), indicating that female patients were more likely to utilize engagement as a coping approach compared to males. A similar trend was noticed in the “Emotion Focused” domain where 59.3% of women scored above the median, contrasting with just 23.5% of men, as shown in Table 4. This vast discrepancy between men and women was highly statistically significant (*p* < 0.001), further emphasizing that women in this cohort were substantially more inclined to employ emotion-focused coping techniques than men.

Female orthognathic patients, when compared to their male counterparts, were found to have a 1.5 times higher risk (HR = 1.5) of depression. This observation was supported by the 95% confidence interval (CI) ranging from 1.1 to 2.0 and was statistically significant with a *p*-value of 0.014. This underscores the idea that being female may be a determinant for heightened depressive symptoms in this specific patient cohort. However, age, on a per-year increase basis, did not demonstrate a substantial link with depression. With an HR of 1.02 and a 95% CI between 0.97 and 1.19, its *p*-value of 0.132 indicated a lack of statistical significance.

Coping mechanisms also emerged as critical factors. Patients who predominantly utilized disengagement as a coping strategy faced a 1.3 time increased risk of depression (*p* = 0.049). Even more pronounced was the link between emotion-focused coping and depression: those resorting to this strategy had a 1.6-fold increased risk, and the association was highly significant with a *p*-value of less than 0.001.

**Figure 3:**
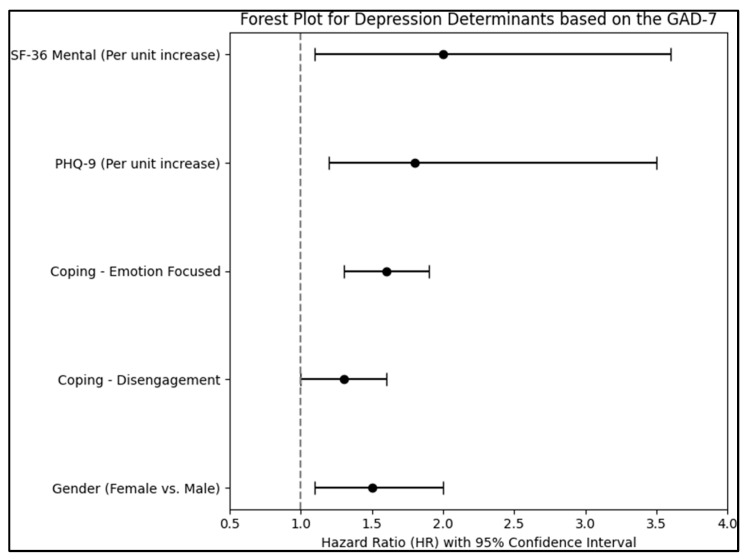
Regression analysis results.

Crucially, increases in PHQ-9 scores, which gauge depression severity, corresponded to a notable 1.8 time increased risk of depression (HR = 1.8; 95% CI: 1.2–3.5), and this link was highly significant (*p* < 0.001). Similarly, for every unit increase in the mental component of the SF-36 scale, which assesses health-related quality of life, the risk for depression doubled (HR = 2.0; 95% CI: 1.1–3.6), a finding reinforced by its *p*-value of less than 0.001. However, the physical component of the SF-36 scale did not exhibit a significant correlation with depression (*p* = 0.086), as presented in Table 5 and Figure 3.

## 4. Discussion

### 4.1. Important Findings and Literature Review

The current study results unveiled intriguing patterns and associations which may shed light on the complex interplay between patient’s sex and psychological determinants in this specific demographic. One of the most striking observations was the significant difference between men and women in perceived mental health status. Women in the cohort consistently reported poorer mental health scores compared to men. This aligns with the broader literature, wherein women often demonstrate a higher prevalence of mood and anxiety disorders compared to men. Interestingly, the SF-36 survey highlighted that while men perceived their mental health to be generally better, there was no significant difference in the overall health-related quality of life between sexes. This suggests that even though women may be experiencing heightened symptoms of anxiety or depression, they do not necessarily translate into an overall reduced quality of life in comparison to their male counterparts.

The GAD-7 and PHQ-9 surveys further supported this narrative, showcasing that female orthognathic patients exhibited more pronounced symptoms of anxiety. While women also tended to score higher on depressive symptoms, the difference was not statistically significant, pointing towards a potential area for future exploration. These findings seem consistent with the established literature where women are often found to be more susceptible to anxiety and mood disorders [9,25]. It is essential to understand the root cause of this predisposition, be it societal, hormonal, or a combination of myriad factors, to craft appropriate interventions.

Coping mechanisms, and more specifically the ways in which they differ between sexes, emerged as another pivotal aspect of our study. Women were found to be considerably more inclined towards emotion-focused coping, while men leaned towards disengagement [26,27]. The inclination of women toward emotion-focused coping is in line with some psychological theories which posit that women may generally be more emotionally expressive and tend to process emotions through discussion or rumination. Conversely, the observed male preference for disengagement, a form of avoidant coping, echoes the stereotypical notion of men being more reticent or avoiding confrontation with emotional challenges.

Another vital finding of our research was the link between coping strategies and depression. Emotion-focused coping was strongly associated with increased depressive symptoms. The close relationship between emotion-focused coping and depression has been documented in other studies, suggesting that while processing emotions can be therapeutic, excessive rumination without actionable problem-solving can exacerbate depressive feelings. On the other hand, disengagement also showed an association, though less profound. This is consistent with the understanding that avoidant coping strategies can lead to unaddressed emotional build-up, further contributing to depressive symptoms [28,29].

Moreover, regarding the regression analysis, the elevated risk of depression in females, as captured by the HR of 1.5, further emphasizes the need for sex-tailored psychological interventions. Simultaneously, other potential determinants such as smoking status and place of origin did not exhibit any significant correlation, suggesting that while these factors might play a role in the general population’s mental health, they might not be as crucial in this specific demographic of orthognathic patients.

In the field of orthognathic research, the role of social support in mitigating pre-surgical anxiety remains underexplored. Notably, one study discerned that only the perceived social support from a significant other, or a “special person”, which correlated with a notable reduction in pre-operative anxiety, while support from family and friends did not significantly influence anxiety levels [30]. This finding hints at the variable effectiveness of different members within a patient’s support network. However, in our study, even though there was a significant proportion of patients that did not have a partner when the surveys were filled, the association of single status was not significantly associated with higher anxiety scores. Consequently, it might be advisable for patients to be accompanied by their primary support person during pre-operative appointments, ensuring this individual receives proper guidance from the clinical team on how best to assist the patient. For those without a distinct support figure, collaborative deliberation between the patient and clinical team could chart ways to bolster necessary support [31].

The study also found that heightened resilience was directly linked to diminished pre-operative anxiety. Although the resilience score in this study lagged slightly behind the general US population’s median, it aligned with scores observed in various UK groups, including university students and older adults. The literature has shed light on a myriad of interventions aimed at augmenting resilience [32], but their efficacy for orthognathic patients remains uncertain, indicating potential avenues for future research. Conversely, the employment of avoidance coping strategies, evasive behaviors to elude confronting stressors, like sidestepping surgery-related questions, was correlated with a surge in anxiety. Interestingly, while past studies have suggested that avoidance coping could actually curb pre-operative anxiety [33,34], the timing of these assessments, often just before surgery, might account for divergent findings. This study’s participants filled out questionnaires up to six weeks prior to surgery, possibly indicating their anxiety and coping methods were still in formation and not yet fully reflective of their imminent surgical state, as reported in other studies [35].

In interpreting the gender differences observed in our study, we found that women reported higher scores on the SF-36 scale, indicating a greater burden of mental health issues compared to men. Additionally, our analysis suggests that women are more inclined towards emotion-focused coping strategies, whereas men more frequently engage in avoidance of emotional challenges. This dichotomy in coping mechanisms underscores the necessity for gender-tailored psychological support in managing mental health. While our findings are indicative, there remains a need to explore the underlying factors contributing to these disparities, such as cultural norms and societal expectations, or biological influences like hormonal variations, to fully understand their implications and to inform more personalized therapeutic interventions [36].

Future studies should explore longitudinal outcomes to assess the durability of psychological impacts post-surgery. Additionally, research expanding beyond binary sex distinctions to include diverse gender identities would offer a more inclusive understanding of patient experiences. Clinically, the findings suggest the need for personalized psychological support tailored to sex-specific risks and coping styles, which could be integrated into preoperative care protocols for orthognathic surgery patients.

### 4.2. Study Limitations

This study, while shedding light on the intricate relationship between men and women, and psychological experiences among orthognathic patients, bears several inherent limitations. First, the cross-sectional nature of the research design restricts our understanding to a particular point in time, potentially overlooking any temporal fluctuations in patient’s psychological experiences leading up to their surgery. The study’s confinement to patients from a single hospital, the Emergency Clinical Municipal Hospital in Timisoara, Romania, raises concerns about its generalizability across different cultural or regional settings. Additionally, the exclusive reliance on self-report instruments, albeit validated, might introduce self-reporting biases, with patients potentially underreporting or overemphasizing their symptoms due to societal expectations or personal beliefs. A limitation of this study is the lack of detailed descriptions of specific depression and anxiety symptoms beyond what was captured using the SF-36, GAD-7, and PHQ-9 questionnaires. While these instruments provide a measure of psychological impact, they may not capture the full spectrum of individual symptomatology experienced by the patients.

The strict inclusion and exclusion criteria, although necessary for study rigor, might limit the study’s applicability to a broader patient demographic, such as those with craniofacial syndromes or who had previously undergone orthognathic treatment. While this study encompasses a wide age range of participants from 18 to 65 years, which introduces variability in the data, we recognize this as a limitation. Moreover, the study was limited to the investigation of biological sex differences (male and female) in relation to anxiety, depressive symptoms, and coping strategies, based on self-reported sex at birth. It did not consider the spectrum of gender identities and the social and cultural constructs associated with them, which represents a limitation in our research. Future studies are encouraged to explore how various gender identities may influence psychological experiences in the context of orthognathic surgery. Lastly, the disparity in sample size, although marginal, between male and female participants may introduce an element of bias in the results, potentially skewing sex-based comparisons.

## 5. Conclusions

This study highlights significant gender-related differences in the psychological impact of orthognathic surgery. Females exhibited higher anxiety and depression levels, as reflected in their PHQ-9 and GAD-7 scores, suggesting a greater vulnerability to these conditions. The research also revealed gender-specific coping mechanisms, with males leaning towards disengagement and females towards engagement and emotion-focused strategies. These findings underscore the need for gender-sensitive approaches in mental health support for orthognathic surgery patients.

## Figures and Tables

**Table 1:** Background characteristics of the study cohort.

Variables	Men (*n* = 68)	Women (*n* = 59)	*p*-Value *
Age, years (mean ± SD) **	32.8 ± 12.5	33.5 ± 14.1	0.767
Obesity (*n*, %)	14 (20.6%)	12 (20.3%)	0.972
Currently smoking	15 (22.1%)	7 (11.9%)	0.130
Alcohol use (occasionally)	23 (33.8%)	18 (30.5%)	0.690
Place of origin (urban)	38 (55.9%)	36 (61.0%)	0.558
Education			
High school	21 (30.9%)	19 (32.2%)	0.880
College	24 (35.3%)	22 (37.3%)	0.815
University	23 (33.8%)	18 (30.5%)	0.724
CCI > 2	6 (8.8%)	4 (6.8%)	0.669
Relationship status (single)	25 (36.8%)	29 (49.2%)	0.159
Unemployed (*n*, %)	11 (16.2%)	8 (13.6%)	0.683
Malocclusion type			0.706
I	10 (14.7%)	11 (18.6%)	
II	33 (48.5%)	30 (50.8%)	
III	25 (36.8%)	18 (30.5%)	
Surgery type			0.651
Single jaw	35 (51.5%)	28 (47.5%)	
Bimaxillary	33 (48.5%)	31 (52.5%)	

* Chi-square or Fisher’s exact test; ** Student’s *t*-test; SD, Standard Deviation; and CCI, Charlson Comorbidity Index.

**Table 2:** Comparison of SF-36 survey results between men and women.

SF-36 (Mean ± SD)	Men (*n* = 68)	Women (*n* = 59)	*p*-Value *
Physical	54.2 ± 7.0	51.5 ± 8.3	0.049
Mental	53.9 ± 6.6	50.2 ± 6.9	0.003
Total score	55.0 ± 8.1	52.6 ± 7.3	0.083

* Student’s *t*-test; SD, standard deviation; SF-36, short form survey (higher scores indicate better health status and quality of life).

**Table 3:** Comparison of GAD-7 and PHQ-9 survey results between men and women.

Variables (Mean ± SD)	Men (*n* = 68)	Women (*n* = 59)	*p*-Value *
GAD-7	5.9 ± 3.3	7.1 ± 3.1	0.037
PHQ-9	4.8 ± 2.5	5.6 ± 2.8	0.091

* Student’s *t*-test; SD, standard deviation; GAD, general anxiety disorder (higher scores indicate higher anxiety symptoms); and PHQ, patient health questionnaire (higher scores indicate more severe depression symptoms).

**Table 4:** Comparison of COPE-60 survey results between men and women.

Variables (% of Scores above Median)	Men (*n* = 68)	Women (*n* = 59)	*p*-Value *
Disengagement	42 (61.8%)	26 (44.1%)	0.046
Engagement	18 (26.5%)	30 (50.8%)	0.004
Emotion Focused	16 (23.5%)	35 (59.3%)	<0.001
Problem Focused	24 (35.3%)	17 (28.8%)	0.435

* Chi-square test; GAD, general anxiety disorder (higher scores indicate higher anxiety symptoms); and COPE, coping orientation to problems experienced inventory (higher scores indicate that patients are more likely to use a certain domain of coping strategies).

**Table 5:** Regression analysis for depression determinants based on the GAD-7.

Independent Variables	HR–Exp(B)	95% CI	*p*-Value
Sex (Female vs. Male)	1.5	1.1–2.0	0.014
Age (Per year increase)	1.02	0.97–1.19	0.132
Coping–Disengagement	1.3	1.0–1.6	0.049
Coping–Emotion Focused	1.6	1.3–1.9	<0.001
Coping–Problem Focused	0.9	0.7–1.1	0.345
Smoking (Yes vs. No)	1.2	0.9–1.6	0.212
Place of origin (Urban)	1.1	0.8–1.4	0.621
Relationship status (Single)	1.3	1.0–1.7	0.056
PHQ-9 (Per unit increase)	1.8	1.2–3.5	<0.001
SF-36 Physical (Per unit increase)	1.1	0.9–1.8	0.086
SF-36 Mental (Per unit increase)	2.0	1.1–3.6	<0.001

HR, hazard ratio; CI, confidence interval; and GAD, generalized anxiety disorder.

## Data Availability

The data presented in this study are available on request from the corresponding author.
